# Smart Phone APP to Restore Optimal Weight (SPAROW): protocol for a randomised controlled trial for women with recent gestational diabetes

**DOI:** 10.1186/s12889-019-7691-3

**Published:** 2019-10-15

**Authors:** Karen Lim, Claudia Chi, Shiao-Yng Chan, Su Lin Lim, Siew Min Ang, Joanne S. Yoong, Cammy Tsai, Su Ren Wong, Tong Wei Yew, E. Shyong Tai, Eu-Leong Yong

**Affiliations:** 1 Department of Obstetrics and Gynecology, National University Hospital, National University of Singapore, Republic of Singapore; 2 Department of Dietetics, National University Hospital, National University of Singapore, Republic of Singapore; 3 Saw Swee Hock School of Public Health, National University of Singapore, Republic of Singapore; 4 Department of Rehabilitation, National University Hospital, National University of Singapore, Republic of Singapore; 5 Department of Medicine, National University Hospital, National University of Singapore, Republic of Singapore

**Keywords:** Randomized controlled trial, Gestational diabetes, Prevention, Smartphone application, Weight loss

## Abstract

**Background:**

Gestational diabetes (GDM) is a known risk factor for type 2 diabetes mellitus (T2DM), and women with a history of GDM have a 7-fold increased risk of developing the disease. Achieving a healthy weight post-delivery is key in reducing the risk of future diabetes in these women. The aim of this trial is to investigate the use of an interactive smartphone application (APP) to restore women to optimal weight following delivery.

**Methods:**

This will be an open-label randomized controlled trial. Two hundred women with gestational diabetes will be randomized to receive the intervention or standard care following delivery. Participants will be reviewed at 6 weeks and 4 months post-delivery. The intervention is an APP serving as a platform for weight, diet and physical activity tracking. The APP provides 3–5 min educational videos suggesting suitable lifestyle adjustments relevant to postnatal period such as breast feeding, diet and exercise. Lastly, the APP will allow real-time interaction between users and the team of dietitians, physiotherapists and occupational therapists to encourage restoration of optimal weight. Women in the control arm will be informed about the increased risk of developing T2DM and advised to maintain a healthy weight. Primary outcome measure is the restoration of participants’ booking weight if booking BMI ≤ 23, or weight loss of at least 5% from booking weight if booking BMI > 23 over the 4 month period. Secondary outcome measures will assess serum metabolic and inflammatory markers, quality of life via questionnaires and cost-effectiveness of the intervention at each follow-up visit.

**Discussion:**

This will be the first randomised controlled trial investigating the use of a smartphone application for postpartum weight loss in women with gestational diabetes. The major ethnic groups in our study population represent the majority of ethnic groups in Asia, amongst which the prevalence of diabetes is high. If shown to be effective, this APP may be used in wider clinical settings to improve postpartum weight loss and reduce the risk of developing T2DM in these women.

**Trial registration:**

This study was registered on clintrials.gov on the 30th of October 2017, under the trial registration number: NCT03324737.

## Background

Globally, over 400 million adults were living with diabetes mellitus in 2014, with age-standardized prevalence of diabetes nearly doubling since 1980 [1]. About half of people with diabetes are estimated to reside in South-East Asia and Western Pacific regions [2]. The annual cost of diabetes worldwide is estimated to be US$1·31 trillion, or 1·8% of the global gross domestic product [3]. Even though patients with Type 2 Diabetes Mellitus (T2DM) have a lower life expectancy, the condition is associated with substantially higher lifetime medical costs. Singapore has one of the highest prevalence of diabetes in Asia (11.3% among adults 18–69 years old) [4]. In Singapore excess lifetime medical expenses for T2DM patients was SGD132,506, to SGD70,110 when the age of T2DM diagnosis was 40 and 65 years, respectively [5]. The lifetime risk of T2DM in Singapore is predicted to be one in two by 2050 [6] prompting the Singapore Ministry of Health to declare a “War on Diabetes” [7].

Gestational diabetes mellitus (GDM) confers a high risk of T2DM, culminating in a 7-fold increased risk with rapid increases in incidence in the first 5 years after delivery [8, 9]. Among women with equivalent degrees of impaired glucose tolerance, those with a history of GDM have 48 and 71% higher risk of developing T2DM when followed-up over 3 and 10 years respectively, compared with those without GDM history [10, 11]. As such, GDM in pregnancy represents a unique opportunity for early intervention in women at risk of subsequent T2DM, well before most community health screening programs for pre-diabetes and type 2 diabetes commence. 20–30% of pregnant women in Singapore are affected by GDM [12, 13], among one of the highest in the world.

Restoring optimal weight following pregnancy is a cost-effective method to reduce incidence of T2DM [14, 15]. In the Diabetes Prevention Program (DPP), diabetes incidence in high-risk adults was reduced by 58% with intensive lifestyle intervention, significantly more effective than treatment with metformin [16]. Among DPP participants with a history of GDM, both intensive lifestyle and metformin are highly effective in preventing diabetes [10, 11]. While there is often ready acceptance of lifestyle changes amongst women during the antenatal period after the diagnosis of GDM, adherence to these lifestyle changes after delivery remains a challenge.

Though some studies that aim to achieve optimal weight after recent GDM using combinations of dietary and physical activity interventions have reported positive outcomes [17, 18], others have found no effects, sustained or otherwise [19, 20]. A study in Australia (with intervention comprising one individual face-to-face session, five group sessions, and two telephone sessions) indicated that although significant weight loss of around 1 kg can be achieved over 12 months with the intervention, only 10% of women attended all sessions, and 34% attended no sessions at all [21]. The main challenge is the delivery of the lifestyle intervention in the most appropriate and acceptable way for women in the postnatal period.

Time constraints were identified as a major barrier to program success [21] and lifestyle interventions were ineffective mainly due to poor in-person attendance of intervention programs by women who just delivered a baby [19, 20, 22]. Alternatives to face-to-face interventions such as correspondence programs [23], and counselling during routine primary care visits [24] have achieved varying successes. Nevertheless post-partum GDM mothers still express strong needs for timely information and social support beyond the home, especially as the combination of excess weight and adjustment to the post-delivery period can result in added stresses and perceived complications [25, 26]. Being part of a social network can influence health behaviour [27]. One mechanism is simply knowledge transfer, which can occur through direct communication or information from an experienced authority or expert. A second mechanism is social influence via role-modelling or norm-setting: individuals compare themselves to socially defined norms or expectations. A third mechanism is the modification of self-efficacy or locus of control due to social support: in the classic social cognitive theory, individuals’ belief in one’s ability to change (i.e. self-efficacy) are critical for behaviour change. Self-efficacy in turn can be positively affected by social support.

There is evidence that mobile technology is acceptable and convenient for a large proportion of pregnant mothers [28]. Leveraging the use of mobile technology in Singapore is particularly promising. Various surveys indicate that Singapore ranks among the world’s most mobile-savvy societies, with a smart-phone penetration rate of 80–90% [29].

Our hypothesis is that a smartphone application (APP) will be effective to restore optimal weight in mothers with recent GDM. The APP will be interactive and incorporate individualized professional lifestyle coaching support, self-monitoring of diet, activity and weight; behaviours shown to be significantly associated with weight loss and maintenance [30, 31, 32]. Localised tips on suitable lifestyle changes, personalized to the local environment will be advocated through short 3–5 min videos. These will be supplemented by physical activity counts and a comprehensive on-line nutritional guide. Through the APP, individualized coaching, feedback and on-line social support will be provided by a team of health and lifestyle coaches including dietitians, physiotherapists and occupational therapists experienced in helping new mothers. The APP will provide holistic support for mothers enabling them to achieve optimal weight and improved cardio-metabolic and inflammatory markers.

To test this hypothesis, we aim to conduct a randomized control trial to examine the efficacy of this APP, customized for Singaporean women with recent GDM, to firstly optimize post-delivery weight compared with standard care. Secondly, to assess the APP’s effect on a panel of markers of cardio-metabolic risk. These secondary outcome data will give indications on women’s future cardiovascular health and capture an underused opportunity to improve women’s health [33]. Thirdly, to evaluate the cost-effectiveness of the APP compared to standard care within the Singapore healthcare system in terms of improvements in quality of life, scalability and sustainability. The SPAROW trial could potentially provide a scalable intervention for promoting healthy lifestyle trajectories with women with recent GDM. If proven effective, the APP will address current challenges in recruitment and retention of GDM mothers in group-based and face-to-face weight-loss interventions and provide data for longer term trials to examine its effectiveness for primary prevention of T2DM in women.

## Methods

This protocol was written using the SPIRIT reporting guidelines [34]. This study will be a randomised controlled trial comparing the APP to standard care in women with recent GDM. Participants will be recruited from women who have recently delivered at the National University Hospital, Singapore (NUH). Each participant will be randomized to the interactive smartphone APP intervention or control standard care arm. In order to understand women’s preferences and to help in the APP design, we conducted a preliminary participant focus group discussion and feasibility study.

### Patient and public involvement

A mixed-methods feasibility study to assess acceptability of a mobile-application based support tool for women with recent GDM was conducted in NUH from May ­ August 2015. A sample of 46 pregnant women who had GDM attending the NUH weekly GDM clinic were surveyed. Results of this survey are shown in Table 1. 90% of those surveyed indicated that they would be interested in some kind of postnatal continued support related to reducing T2DM risk. When asked for the most preferred platform through which information could best be delivered, counter-intuitively only 17% preferred individual counselling and even fewer (14%) indicated group classes as their preferred option for support. Focus group discussions of postnatal women with previous GDM indicated that women post-delivery were busy looking after the new-born, were time-challenged, and their own personal health needs tended to receive the lowest priority. The most popular options were web-based (38%) or smartphone app-based (20%) resources. Since Singapore has a very high (90%) penetration rate for smartphones, we reasoned that our smartphone application with web interaction capabilities would be the most preferred option for some 60% of Singaporean women with recent GDM. When asked what type of information was preferred, most women (67-70%) wanted general tips on lifestyle change and nutritional/activity tracking (70% each), but also some personal monitoring capability. Notably social support (20%) and reminders (9%) ranked relatively lower.

**Table 1:** Results of feasibility survey

Most preferred support platform	% women
Website/ online course	38
Smartphone app	20
Individual counselling	17
Text Messages	15
Group classes	14
Print materials	8
Telephone support	3
Type of information	% answering yes
General tips on lifestyle change	70
Tracking nutrition/physical activity	70
Tracking blood tests and other health outcomes	67
Guidance on lifestyle change	64
Reminders and prompts	36
Social Support	20
Other	9

The results of this feasibility study were taken into account in the design of our clinical trial.

### Design and setting

**Fig. 1:**
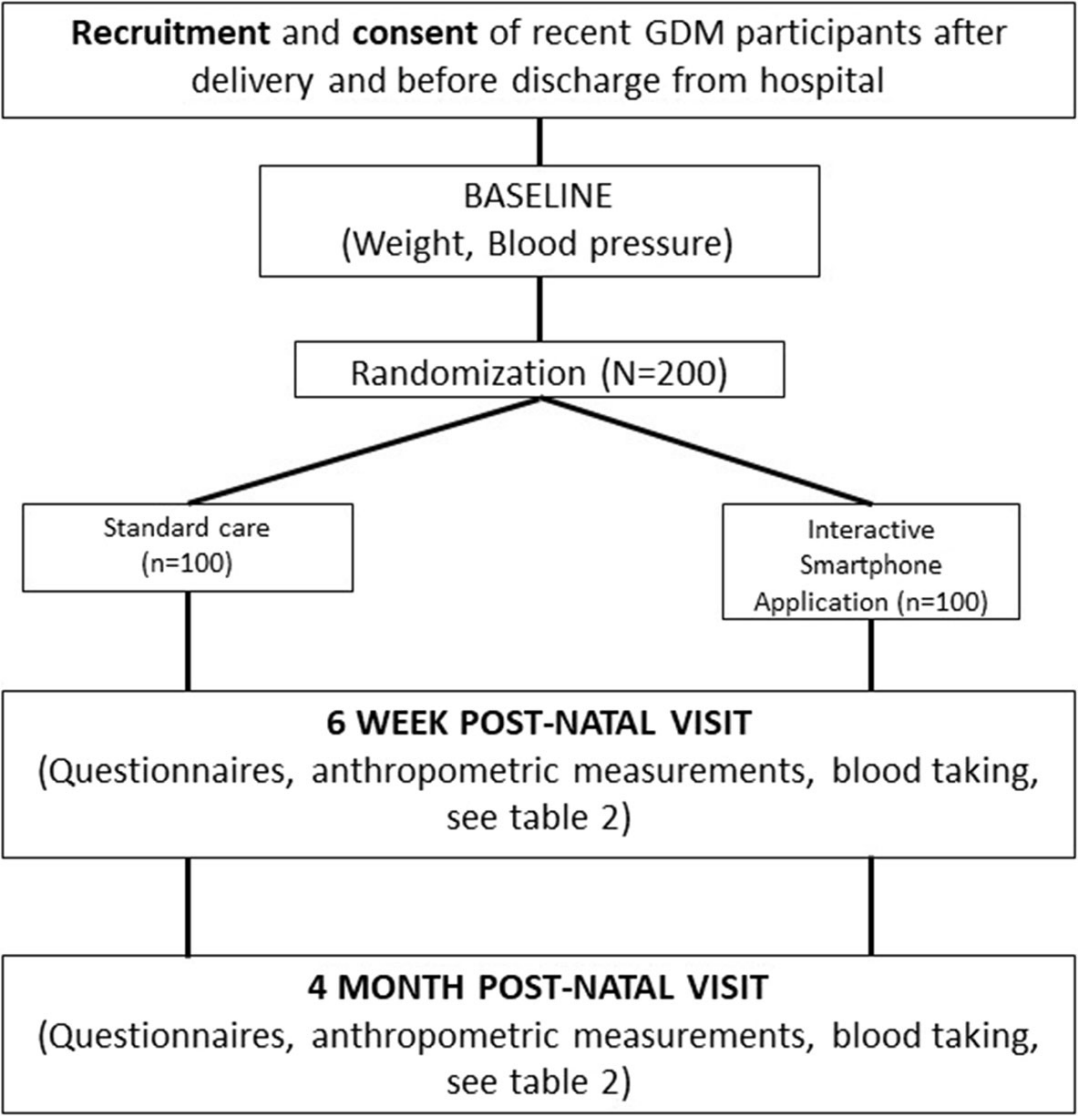
Flow of participants in randomized control trial

The overall study protocol is shown in Fig. 1. The study has been approved by the Domain Specific Review Board of the National Healthcare Group.

### Recruitment

Electronic medical records of women in the postnatal ward will be screened daily by members of the research team to determine eligibility. If eligibility criteria are met, written information in the form of a pamphlet will be provided together with a verbal explanation of what the study entails. Following this, individuals will be given ample time to choose if they will like to participate. If agreeable, written consent will be obtained by designated members of the research team.

### Inclusion criteria

- Age 21 years and above
- Diagnosed with GDM antenatally (between 24 and 34 weeks gestation) defined using the 2013 World Health Organisation criteria: fasting plasma glucose ≥5.1 mmol/L, 1-h plasma glucose ≥10.0 mmol/L and/or 2-h plasma glucose ≥8.5 mmol/L following a 75 g oral glucose load (World Health Organisation, 2013) [34]
- Has a smartphone and able to independently use a smartphone app
- Able to speak and read English
- Preconception or first trimester weight available at/or before 12 weeks

### Exclusion criteria

- Women with pre-existing type 1 or type 2 diabetes mellitus
- Women with a terminal or life-threatening condition, or a physical/mental condition that would prevent completion of a majority of study instruments
- Women whose pregnancy resulted in a preterm delivery before 36 weeks

### Study protocol (Fig. 1)

For eligible subjects, a baseline visit will be conducted in the postnatal ward, during which informed consent will be sought, eligibility formally assessed, and baseline measurements collected. The participants will then be randomised to either the intervention or control arm and participants allocated to the control arm will be given ‘standard care’ which entails a follow-up appointment at 6 weeks postpartum for review by a clinician with routine postnatal check, dietary advice and a repeat oral glucose tolerance test. Women found to have impaired fasting glucose (6.1–6.9 mmol/L) or impaired glucose tolerance (2H post glucose of 7.8–11.0 mmol/L), will be issued a letter reinforcing lifestyle changes namely weight loss (if raised BMI), diet, exercise, and encouragement to consult a family physician to discuss the appropriateness of starting medications that can prevent the progression to type 2 diabetes, or restore the blood glucose to normal levels. Participants found to have diabetes (fasting > 7 mmol, or 2H > 11 mmol/L) will be referred to their family physician or internist of their choice. Women with a normal OGTT will be informed of their normal result. Women allocated to the intervention arm will be asked to download the smart phone APP and briefed on its use, in addition to receiving the standard care as mentioned above. Both participants in the control and intervention arm will be followed up at 6 weeks and 4 months postnatal, during which anthropometric measurements, blood tests and questionnaires will be performed (List of investigations, Table 2).

**Table 2:** List of investigations

Domains	Baseline	Outcome visits (1 & 2)
Self-Administered Questionnaires		3-day food diary
		Self-Efficacy to regulate eating habits
		Self-Efficacy to regulate exercise
		Health Education Impact (heiQ)
		Quality of Life (RAND-12)
		Health Expenditure
Biophysical	Height and weight	Height and weight
	Blood pressure	Waist circumference
		Blood pressure
Physical Performance		Right hand grip strength
Breastfeeding Status		Fully, mixed or no breastfeeding
Blood sample collection		75 g 2-h oral glucose tolerance test
		HbA1c
		Advanced glycation end-products
		C-peptide
		HOMA-IR
		Lipid profile
		Liver function
		High sensitivity C-reactive protein
		Interleukin-6

### Randomization and enrolment

Participants will be randomized at the baseline visit to the intervention or control arm, using a permuted block design randomisation with blocks of 4. An independent researcher will generate the set of sequences and assign participants to the intervention groups using sequentially numbered sealed opaque envelopes to ensure allocation concealment until interventions are assigned. Due to the nature of the intervention, blinding of participants and assessors was not possible.

### Intervention arm – nBuddy smartphone APP

The Nutritionist Buddy (nBuddy) APP was developed using the Obesity-Related Behavioural Intervention Trials (ORBIT) Model for behavioural treatment as a framework for translating behavioural science discoveries into treatments, as it is a flexible and robust process to design, conduct and evaluate mobile technology-based behavioural interventions [35, 36]. The platform was adapted for women with recent GDM, with the primary aim of restoring optimal weight postpartum in a timely manner. We hypothesized a pathway by which the behavioural treatment could solve clinical problems related to lifestyle, articulate the clinically significant milestones targeted, and define potential treatment strategies to help subjects achieve these milestones. The APP has several on-line features to facilitate this, including tracking tools for diet, exercise, and progress reports, and supports real-time interaction between users and health and lifestyle coaches. There is also a backend system for the investigators to monitor their progress. Furthermore educational information, such as videos can also be pushed to individuals, making the user’s experience on the APP a personalised one.

There are five main components to the APP:

#### Goal setting

The APP will personalize diet, activity and weight regulation targets based on their baseline weight at study entry versus optimal post-delivery weight. Calorie and exercise goals will be gradually changed based on the performance of the participant every week. Tools for change such as encouraging positive internal and external motivations, exploring self-efficacy, increasing readiness to change, active ownership of their health, exploring resources and barriers to a healthy lifestyle (environmental, personal) and setting SMART personal goals [37, 38] will be explored with patient at the beginning of the intervention and dynamically through the on-line coaching feature of the APP.

#### Food choices and recommendations

The APP consists of a nutritional database of more than 11,000 local foods, including tradionally recommended or ‘confinemnet foods’ of the main ethnicities in Singapore (Chinese, Malay and Indian). With this extensive database, the APP has a built-in automated and immediate recommendation of foods if the choice selected is not the best choice for the participant. These healthier food recommendations will be based on the foods selected taking into consideration the ethnicity of the participant. Calorie and nutrient trackers will be incorporated to enable participants to monitor their nutrition. There will be prompts to remind participants if they have exceeded their calorie limit for the meal, or for the day. Graphical reports will be available for users to track their daily calorie intake and compare against their targets.

#### Activity and steps tracker

The APP has a built-in pedometer to count physical activity steps to enable patients to track their progress towards program goals in a seamless and effective manner. In-addition, users also have the option to input the type of activities, such as gardening, sweeping and mopping, which will translate automatically into step counts. Graphical reports will be available for the users to track their number of daily steps to compare against their targets.

#### Interactive video lessons

The APP will have 16 video lessons which are specially commissioned for the particular needs of women with recent GDM. The videos encompass various aspects of diet, lifestyle and behaviour management including:

- Emotional health for new mothers,
- Breastfeeding,
- Diet for new mothers
- Exercises for postnatal women
- Reducing the risk of getting diabetes
- Weaning diet for babies.

These videos have been specially made to last about 3 min to hold the attention of busy mothers. Videos will be pushed out according to the needs of the particular participant.

#### On-line lifestyle support and coaching

The APP has a chat channel to enable live interaction between the participants and the study team of dietitians, physiotherapists, occupational therapists. Clinical issues brought up will be brought to the attention of relevant clinicians and appropriate follow-up care arranged. For the study team, there will be a real-time dashboard to monitor and track participant’s diet intake, food choices, activity levels/ type of exercise undertaken, and weight with alerts to intervene as required. Adjustment strategies to the challenges of motherhood (and returning to work) will be emphasized. Reflections on self-care behaviours (healthy eating, staying active, taking charge, problem solving, reducing modifiable risk factors, and healthy emotions) will be encouraged. Psycho-social aspects of transition to motherhood including addressing guilt and other unhelpful emotions, managing breast-feeding, optimizing routines for healthy eating and physical activity, moving beyond set-backs and problem-solving will be communicated on-line according to the needs of the participant [39]. Common musculoskeletal issues, such as aches and pains, queries on returning to certain sports or activities will also be addressed individually on the dashboard, to further support adherence.

### Outcomes

Outcomes will be measured at 6 weeks and 4 months postpartum.

#### Primary

The primary outcome measure of this study will be restoration of booking weight at 4 months postpartum if previous booking BMI ≤23, and weight loss of at least 5% with respect to booking weight if BMI > 23.

#### Secondary

Quantitative measures of markers predictive of future type 2 diabetes mellitus and cardio-metabolic risk will be performed. Absolute weight loss between groups will be measured and compared. Breastfeeding status will be recorded, as fully, partial or no breastfeeding. Anthropometric measurements in addition to BMI will be assessed, such as right-hand grip strength, and waist circumference. Questionnaires will be used to assess quality of life, self-efficacy, health behaviours and dietary choices. Analyses on cost effectiveness will be performed at the end of the study.

#### Exploratory outcomes

- Percentage of participants able to achieve restoration of booking weight or less if BMI ≤ 23, or weight loss of at least 5% of booking weight if BMI > 23 at 6 weeks and 4 months postnatal
- Percentage of participants diagnosed with impaired glucose tolerance or T2DM at 6 weeks and 4 months postnatal oral glucose tolerance test.
- Difference between weight, BMI, waist circumference, and right hand grip strength at 6 weeks and 4 months postnatal.
- Difference in serum markers for future T2DM (see Table 2: List of investigations) at 6 weeks and 4 months postnatal
- Change in questionnaire scores (Table 2) at 6 weeks and 4 months postnatal
- Quality of life and cost-effectiveness analysis

### Statistics and sample size

For the main analysis, the primary outcomes are the proportion of women attaining a satisfactory weight (as defined above) at 4 months post-delivery. We will aim for a minimally important effect of a doubling in the proportion of women who attain their target weight, corresponding to 40% of women in the intervention group attaining their target weight (or less) and 20% in the control group. For an alpha of 0.05, with a potential 15% attrition rate, we would need to recruit 75 individuals in each group to achieve power of 80%, or 104 individuals for a power of 90%. Therefore we aim to recruit 100 individuals in each group. Rationale: Based on the outcomes for intervention and control groups in a telephone-based intervention study [23] about 23% of the control group achieved target weights compared with 30% in the intervention arm. We propose that the effect size of our intervention will be higher as there is evidence that more intensive weight measurements and interactive elements exhibit greater effectiveness [23].

### Data collection

Patient information will be collected on in a de-identified manner and hard copy forms will be stored in locked cabinets accessible only by team members. Electronic data will be stored on a secured computer that is password-protected. The databases will not contain subject identifiers and the data linking subject identifiers and the subject identification codes will be stored separately.

### Primary analysis

The primary outcome measure of this study will be restoration of booking weight at 4 months postpartum if previous booking BMI ≤23, and weight loss of at least 5% with respect to booking weight if BMI > 23.

The primary analysis will be performed on the intention-to-treat dataset (all randomised participants, whether they have received the intervention or not, who have provided a weight at 4 months (± 4 weeks) post-delivery) and will be an odds ratio (OR, 95%CI) of the % of women who return to booking weight or less if previous booking BMI ≤23, or achieved weight loss of at least 5% with respect to booking weight (kg to 1 decimal place) if booking BMI > 23. The accuracy of Seca 799 weight machine used was 50 g for < 50 kg and 100 g for 50–150 kg.

Booking weight was defined as booking weight before first pregnancy visit or weight at first trimester closest to 12 weeks but before 13 weeks of completed gestation.

Subsequent models will additionally be adjusted using logistic regression for factors thought to be associated with post-delivery weight loss: ethnicity and parity .

No interim analysis will be performed. Analysis of the primary outcome will occur after all participants have completed all study visits and after all data to that point have been entered, validated and locked.

Tables will be presented showing the characteristics of a) all recruited women, b) all who provided primary outcome, c) all withdrawn before primary outcome.

### Secondary outcomes

Analysis of differences between intervention and control groups will be performed for:

1. Absolute weight change as a continuous variable between the following time points using repeated measures testing, to compare change in weight over the 4 month period between intervention and control using a linear mixed model adjusting for baseline weight.
2. Similarly, for the other secondary outcomes which are continuous, we will compare the effect of intervention over the 4 month period using a linear mixed model adjusting for baseline weight. For non-continuous variables such as breastfeeding status (exclusive or partial/none), a mixed effects logistic regression to evaluate the effect of intervention over the 4 month period.

### Protocol deviations

Any deviations from the protocol, withdrawals or non-compliance to assigned intervention will be recorded, with reasons provided.

### Cost effectiveness analysis

We will also aim to understand if using the interactive APP can be cost-effective relative to standard care (comparing the treatment to the control group), within the Singapore context. Cost-effectiveness analyses will be conducted from the perspective of the healthcare system (direct intervention costs only). We will also evaluate the feasibility of measuring the direct and indirect costs incurred by the participants, including food costs and the costs of additional equipment purchased or activities voluntarily undertaken, as the collection (especially food costs) may raise the burden of participation and depress study take-up, while yielding relatively low quality data. If found to be feasible, we will also undertake to measure cost-effectiveness from a societal perspective.

### Retention and withdrawal

Each participant will have the right to drop out of the study at any point. Data collected up to the point at which the participant withdraws will be used for analysis unless they request otherwise. The reason for participant withdrawal will be recorded in their case report form (CRF) and reported using the CONSORT diagram. The personalized coaching element of the intervention aims to increase retention rates as study team members are able to communicate with participants and explore reasons for choosing to drop out. Participants will be reimbursed monetarily for their time taken off from caring for their child and/or work.

## Discussion

To our knowledge, this will be the first randomised controlled trial investigating the use of a smartphone application for postpartum weight loss in women with gestational diabetes. In contrast to studies which relied on postpartum weight measurements obtained from electronic health records, participants in our study will be asked to return for precise anthropometric and functional measurements by trained research team members following a precise protocol. If proven to be effective in this selected cohort, our findings are likely to be scalable to the entire Singapore population, as the penertration of smartphone use in the island is 80-90%. The intervention includes several mechanisms targeted at achieving weight loss, such as personal goal setting, food choices and recommendations, activity and steps tracking, interactive video lessons and online lifestyle support and coaching. If the APP proves to be effective in restoring optimal weight, further analyses will provide information on which specific mechanism of the interactive smartphone application is most effective.

A limitation is that our subjects will be recruited from a single maternity hospital in Singapore. Nevertheless, the major ethnic groups in our study (Chinese, Malays and Indians) are also the major ethnic groups in Asia. Therefore, interventions in this study, if proven to be effective, may be applicable to the wider Asian population. Another limitation is that the intervention relies on the real-time interaction between participants and the research team, which may affect scalability as this is costly in terms of manpower requrements. We will evaluate the feasibility of the real-time interaction component of our intervention in our cost-effective analysis.

## Data Availability

The datasets generated and/or analysed during the current study are not publicly available as data is currently still being analysed but are available from the corresponding author on reasonable request.

## Abbreviations

APP: Smartphone application
CRF: Case report form
DPP: Diabetes prevention program
GDM: Gestational diabetes mellitus
nBuddy: Nutritionist buddy
NUH: National University Hospital
ORBIT: Obesity-Related Behavioural Intervention Trials
T2DM: Type 2 diabetes mellitus
